# Annual acknowledgement of reviewers

**DOI:** 10.1186/1471-2350-15-20

**Published:** 2014-01-30

**Authors:** Timothy R Sands

**Affiliations:** 1 BioMed Central, Floor 6, 236 Gray's Inn Road, London WC1X 8HB, UK

## Abstract

**Contributing reviewers:**

The editors of BMC Medical Genetics would like to thank all our reviewers who have contributed to the journal in Volume 14 (2013).

## 

Amir Abbasi

Pakistan

Jose Abdenur

USA

John Achermann

UK

Margaret Adam

USA

Ilir Agalliu

USA

Arvind Ahuja

India

Masashi Akiyama

Japan

Ahmed Al Saleh

Saudi Arabia

Fowzan Alkuraya

Saudi Arabia

Valerie Allamand

France

Seth Alper

USA

Gregor Andelfinger

Canada

Miguel Andrade-Navarro

Germany

Ivan Angulo

Brazil

Muhammad Ansar

Pakistan

Anthony Antonellis

USA

Paolo Aretini

Italy

Tadao Arinami

Japan

Rector Arya

USA

Stella Aslibekyan

USA

Halil Ibrahim Atasoy

Turkey

Michaela Auer-Grumbach

Austria

Mona Awadalla

Australia

Hideo Baba

Japan

Elena Bacchelli

Italy

Claudia Bagni

Belgium

Pantelis Bagos

Greece

Ana Bakija-Konsuo

Croatia

Sanjay Kumar Banerjee

India

Vladislav Baranov

Russia

Cynthia Bartels

USA

Jasmin Bartl

Switzerland

Daniel Batlle

USA

Tara Beattie

Canada

Britt Maria Beckmann

Germany

Alessandro Beghini

Italy

Elijah Behr

UK

Tawfeg Ben-Omran

Qatar

Conceicao Bettencourt

UK

Eric Beyer

USA

Amarjit Bhanwer

India

Harald Binder

Germany

Kelly Birdwell

USA

Ralf Birkenhäger

Germany

Peyman Björklund

Sweden

Nevado Blanco

Spain

Luigi Boccuto

USA

Jolanda Boer

Netherlands

Carsten Böger

Germany

Anna Rita Bonfigli

Italy

Nabila Bouatia-Naji

France

Anna Boyajyan

Armenia

Bernard Brais

Canada

Francesco Brancati

Italy

Raja Brauner

France

Jerome Brody

USA

Lesley Bruce

UK

Nicola Brunetti-Pierri

Italy

David Bunyan

UK

Adam Butterworth

UK

Carmen Cadilla

USA

Maria A Caligo

Italy

Lisa Cameron

Canada

Federico Canzian

Germany

Nicola Carboni

Jamaica

Melissa Carter

Canada

Sergi Castellvi-Bel

Spain

John Castiblanco

USA

Weichiao Chang

Taiwan

Chia-Hsiang Chen

Taiwan

Ji Yih Chen

Taiwan

Rong Chen

USA

Zhuo Chen

USA

Yu-Ching Cheng

USA

Pietro Chiurazzi

Italy

Murim Choi

USA

Sven Cichon

Switzerland

Marina Colombi

Italy

Lawreen H Connors

USA

Fabio Coppedè

Italy

Marta Cordoba

Argentina

Aiden Corvin

Ireland

Harriet Corvol

France

Gregory Costain

Canada

Frans Cremers

Netherlands

Julio Croda

Brazil

Sergio Cuevas-Covarrubias

Mexico

Hengmi Cui

China

Robert Culverhouse

USA

Francesca Dagna

Italy

Neetu Dahiya

USA

Pu Dai

China

Denise Daley

USA

Bruno Dallapiccola

Italy

Saeed Daneshmandi

Iran

Ignacio Davila

Spain

Camila Andréa De Oliveira

Brazil

Marina De Rosa

Italy

George Dedoussis

Greece

Ignacio del Castillo

Spain

Marcela del Rio

Spain

Ralph Delfino

USA

Jesús Delgado Calle

Spain

Charles DeLisi

USA

Masashi Demura

Japan

Marcella Devoto

USA

Oscar Diaz Horta

USA

Haner Direskeneli

Turkey

Steven Dobrowolski

USA

James Dowling

USA

Elise Drouin

USA

Denis Duboc

France

Mathieu Emily

France

Sean Ennis

Ireland

Gregory Enns

USA

Raul Estevez

Spain

Agustin Fernandez

Spain

Robert Ferrell

USA

Gianluigi Forloni

Italy

Renato Franco

Italy

Alexis Frazier-Wood

USA

Hudson Freeze

USA

Tomomi Fujisawa

Japan

John Fyfe

USA

Xin Gao

China

Valentina Gatta

Italy

Katarzyna Gaweda-Walerych

Poland

Massimo Gennarelli

Italy

Ivan Gentile

Italy

Maurizio Genuardi

Italy

Philip Giampietro

USA

Michael Gill

Ireland

Hans Goebel

Germany

Celso Gomez-Sanchez

USA

Margarida Gonçalo

Portugal

Marilda Goncalves

Brazil

Miguel Gonzalez-Gay

Spain

Ivan Gorlov

USA

Grazyna Gromadzka

Poland

Renzo Guerrini

Italy

Xiong Guo

China

Fiorella Gurrieri

Italy

Christiane Haase

Denmark

Andreas Hadjisavvas

Cyprus

Yaling Han

China

Dana Hancock

USA

Grahame Hardie

UK

Muhammad Hassan

Pakistan

Michael Hauser

USA

Karen Heath

Spain

Andreas Hector

Germany

Ken-Ichi Hirano

Japan

Yuji Hiromatsu

Japan

Conrad Hodgkinson

USA

Julia Hoefele

Germany

Linda Holmquist Mengelbier

Sweden

Yukio Horikawa

Japan

Laura Horsfall

UK

Chia-Wen Hsu

USA

Whady Hueb

Brazil

Maria Hughes

UK

Maria Hurle

Spain

Mohammad Ilyas

UK

Naoko Iwasaki

Japan

David Jeffries

Gambia

Johan Jocken

Netherlands

Jill Johnsen

USA

Philippe Joly

France

Virginia Kaklamani

USA

Anna Kaminska

Poland

Nahoko Kaniwa

Japan

Michael Karampelas

UK

Fiona Karet

UK

Norihiro Kato

Japan

Tomohiro Katsuya

Japan

David Kaye

Australia

Chiea Chuen Khor

Singapore

Madhu Khullar

India

Yoshiaki Kikkawa

Japan

Jae-Min Kim

South Korea

Toru Kimura

Japan

Anton Kiselev

Russia

Joakim Klar

Sweden

Michael Klintschar

Germany

Julian Knight

UK

Kevin Knower

Australia

Lester Kobzik

USA

Carsten Korth

Germany

Ian Krantz

USA

Marcin Krawczyk

Germany

Gajanan Kulkarni

Canada

Kung-Kai Kuo

Taiwan

Mateusz Kurzawski

Poland

Wenli Lai

China

Nicoletta Landsberger

Italy

Dominique Langin

France

Helen Latsoudis

Greece

Ben Li

USA

Dar-Shong Lin

Taiwan

Xie Li-Ping

China

Augusto Litonjua

USA

Alberto Loche

Switzerland

Kerstin Ludwig

Germany

Carol MacArthur

USA

Fiona Macdonald

UK

Alastair MacLennan

Australia

Ikuhiro Maeda

Japan

Shiro Maeda

Japan

Shinya Maekawa

Japan

Elena Maestrini

Italy

Nejat Mahdieh

Iran

Anurupa Maitra

India

Shohreh Maleki

Sweden

Giovanni Malerba

Italy

Michelangelo Mancuso

Italy

Valentina Mangano

Italy

Minna Mannikko

Finland

Vanessa Suñé Mattevi

Brazil

Ross McManus

Ireland

Kim McBride

USA

Jacob McCauley

USA

Andrew McIntosh

UK

Carolina Medina-Gomez

Netherlands

Monica Melo

Brazil

Phillip Melton

Australia

Claudia Menzaghi

Italy

Nuala Meyer

USA

Maria Giuseppina Miano

Italy

Anne-Marie Minihane

UK

Prasun Mishra

USA

Takeshi Miyamoto

Japan

Emna Mkaouar-Rebai

Tunisia

Maja Mockenhaupt

Germany

Pooneh Mokarram

Iran

Monika Morak

Germany

Miguel Moreno-Pelayo

Spain

Rodrigo Moreno-Reyes

Belgium

Derek Morris

Ireland

Umberto Moscato

Italy

Bertram Müller-Myhsok

Germany

Jan-Willem Muntjewerff

Netherlands

Muhammad Naeem

Pakistan

Masanori Nakagawa

Japan

Koichi Nakanishi

Japan

Esther Nederhof

Netherlands

Giovanni Neri

Italy

Grethe Neumann Andersen

Denmark

Maggie Ng

USA

Vladyslav Nikolayevskyy

UK

Georgios Nikolopoulos

Greece

Cora O'Neill

Ireland

Javier Gonzalo Ocejo-Vinyals

Spain

Bruno Oertel

Germany

Antonio Oliva

Italy

Ken Ong

UK

Alfredo Orrico

Italy

Haruhiko Osawa

Japan

Yuzuru Otsuka

Japan

Cees Oudejans

Netherlands

Mustafa Ozen

Turkey

Sanna Pakkanen

Finland

Barry Palmer

New Zealand

Laura Papi

Italy

Jong Hoon Park

South Korea

Anne Parle-McDermott

Ireland

Eric Pasmant

France

Guglielmina Pepe

Italy

Jose-Luis Perez-Castrillon

Spain

Carolina Perez-Iratxeta

Canada

Tom Pettersson

Finland

Vito Pistoia

Italy

Alessandra Pontillo

Brazil

Ludmila Prokunina-Olsson

USA

Man Qin

China

Jianhua Qiu

China

Sivaramakrishna Rachakonda

Germany

Mangalathu Rajeevan

USA

Michele Ramsay

South Africa

Sajid Rashid

Pakistan

Umbertina Reed

Brazil

Alessandra Renieri

Italy

William Rizzo

USA

Lisa Roberts

South Africa

Sébastien Robiou Du Pont

Canada

Ghislain Rocheleau

Canada

Laia Rodriguez-Revenga

Spain

Angela Rogers

USA

Karen Rouault

France

Victor Ruiz-Perez

Spain

Michael Bjørn Russell

Norway

Malgorzata Rydzanicz

Poland

Khashayar Sakhaee

USA

Towhid Salam

USA

Federica Sangiuolo

Italy

Nobuo Sanjo

Japan

Regie Lyn Santos-Cortez

USA

Richa Saxena

USA

Fernando Scaglia

USA

André Scherag

Germany

Albert Schinzel

Switzerland

Gudrun Schleiermacher

France

Markus Schubert

Germany

John Scott

Australia

Suma Shankar

USA

Makiko Shimizu

Japan

Marwan Shinawi

USA

Oliver Sieber

Australia

Jean-Pierre Siffroi

France

Dheer Singh

India

Anne Slavotinek

USA

Hugo Sousa

Portugal

Melissa Southey

Australia

Vivi Srivastava

India

Frauke Stanke

Germany

Pawel Stankiewicz

USA

Mouna Stayoussef

Tunisia

Martin Steinberg

USA

Blanka Stiburkova

Czech Republic

Liborio Stuppia

Italy

Masaya Sugiyama

Japan

Pierre Szepetowski

France

Hiroshi Takashima

Japan

Yasuhiro Takeshima

Japan

Tiong Yang Tan

Australia

Muhammad Tariq

USA

Wichittra Tassaneeyakul

Thailand

Alexander Malcolm Taylor

UK

Rob Taylor

UK

Maria Tejero

Mexico

Mustafa Tekin

USA

Jacob Tfelt-Hansen

Denmark

Per Medbøe Thorsby

Norway

Francesco Danilo Tiziano

Italy

Akemi Tomoda

Japan

Léon-Charles Tranchevent

Belgium

Lisbeth Tranebjaerg

Denmark

Daniela Turchetti

Italy

Benjamin Tycko

USA

Ching-Cherng Tzeng

Taiwan

Korkut Ulucan

Turkey

Philippe Vago

France

Marie Van Dijk

Netherlands

Mathilde Varret

France

Cecilia Vecoli

Italy

Patricia Veras

Brazil

Chris Verschoor

Canada

Sebastien Viatte

UK

Alessandra Viel

Italy

Karani Santhanakrishnan Vimaleswaran

UK

Tatyana Vlaykova

Bulgaria

Venkatasaroja Voruganti

USA

Raimund Wagener

Germany

Tom Walsh

USA

Lei Wan

Taiwan

Hongwei Wang

China

Philine Wangemann

USA

Richard Ward

USA

Nicole Weisschuh

Germany

Bo Wen

China

Marquitta White

USA

Claus Wilke

USA

Nigel Williams

UK

Judy Wong

Canada

Lee-Jun Wong

USA

Bo Xi

China

Sheng Xiang Xiao

China

Xiaolei Xu

USA

Masahito Yamada

Japan

Hidehisa Yamagata

Japan

Kimiko Yamakawa-Kobayashi

Japan

Toshiyuki Yamamoto

Japan

Shun-Fa Yang

Taiwan

Shea Ping Yip

China

Koh-Ichiro Yoshiura

Japan

Feng Yue

USA

Tanja Zeller

Germany

Maria-Christina Zennaro

France

Kui Zhang

USA

Hua Zhao

USA

Qi Zhao

USA

Wenlei Zhuo

China

Malek Zihlif

Jordan

Peter Zill

Germany

Elias Zintzaras

Greece

Marcella Zollino

Italy

Gabor Zsurka

Germany

## Pre-publication history

The pre-publication history for this paper can be accessed here:

http://www.biomedcentral.com/1471-2350/15/20/prepub

